# Racial differences in a high-risk colorectal cancer referral population: a single-center experience

**DOI:** 10.1186/1897-4287-9-S1-P15

**Published:** 2011-03-10

**Authors:** Cassandra Gulden, Tovah Moss, Olufunmilayo I Olopade, Sonia Kupfer

**Affiliations:** 1 Cancer Risk Clinic, Section of Hematology/Oncology, University of Chicago Medical Center, Chicago, IL, 60637, USA; 2 Section of Gastroenterology, University of Chicago Medical Center, Chicago, IL, 60637, USA

## Background

Multiple studies have shown that colorectal cancer (CRC) disparities exist between African Americans and Caucasians. African Americans have higher CRC incidence and mortality than Caucasians [1,2]. Furthermore, African Americans present with more advanced CRC [3] and at younger ages [4]. However, little is known about the impact of hereditary CRC syndromes in African Americans on these disparities. In the University of Chicago Cancer Risk Clinic, we see a diverse referral population for evaluation of hereditary syndromes based on personal or family history of CRC. We sought to compare clinical characteristics of our African American and Caucasian patients to determine if differences exist in a high-risk CRC referral population.

## Method

We retrospectively collected data on self-reported African American and Caucasian individuals who were referred for a personal or family history of CRC from 1992-2010. Available clinical characteristics including number of individuals, number of families (probands with family members who followed-up in our clinic), personal or family history of CRC, age at cancer onset, and number of individuals who underwent genetic testing for *APC* or mismatch repair genes were collected. Pedigrees were reviewed to determine Revised Bethesda, Amsterdam II and PREMM1,2 scores. Statistical analysis included Student’s t-test for continuous variables and Pearson’s chi-square for categorical variables. Comparison of independent proportions was done using z-ratios.

## Results

A total of 949 Caucasian and 254 African American individuals were referred to the Cancer Risk Clinic for a personal or family history of CRC. The average age of CRC diagnosis, number of family members with CRC, and those meeting Bethesda and Amsterdam criteria were similar between populations (Table 1). Fewer family members of African American probands were seen compared to Caucasians (p =0.002). More African American females with CRC were seen in our clinic (p=0.04). Importantly, despite overall similar clinical characteristics, fewer African Americans had genetic testing compared to Caucasians (p = 0.02).

**Table 1:** Clinical characteristics of individuals referred for personal and/or family history of CRC by race

	AA	Caucasian	p-value
**Personal and/or family history of CRC**			

Total number of individuals	254	949	
Total number families (% of total individuals)	68 (26.8)	617 (65.0)	0.002
Median # CRC affected family members (range)	1 (1-7)	1 (1-14)	
Average number CRC affected family members < 50 years	0.35	0.46	
Number with polyps (%)	6 (2.4)	43 (4.5)	0.12
Genetic testing (%)	16 (6.3)	106 (11.2)	0.02

**Personal History of CRC**			

Total number (% of total)	32 (12.6)	86 (9.1)	0.09
Age years (mean)	48.6	49.9	0.65
% female	82.1	60.5	0.04
Revised Bethesda + (%)	22 (68.8)	49 (57.0)	0.25
Amsterdam II + (%)	5 (15.6)	17 (19.8)	0.61
PREMM1,2 score	17.06	16.12	0.81
Genetic testing (%)	10 (31.2)	42 (48.8)	0.09

CRC, colorectal cancer; AA, African American; Genetic testing category includes mismatch repair genes and *APC;* PREMM1,2 score obtained using publicly available calculator

## Conclusion

Despite many similarities between our high-risk CRC populations, there exists a difference in the number of family members of African American probands seen in our clinic and the number of African Americans who had genetic testing when compared to Caucasians. Less genetic testing may mean African American mutation carriers are not being identified. This can significantly impact appropriate cancer screening recommendations and knowledge about hereditary CRC syndromes in this population. Future collaborative studies are needed to address why these differences may exist.
